# Atrial Fibrillation Detection with Single-Lead Electrocardiogram Based on Temporal Convolutional Network–ResNet

**DOI:** 10.3390/s24020398

**Published:** 2024-01-09

**Authors:** Xiangyu Zhao, Rong Zhou, Li Ning, Qiuquan Guo, Yan Liang, Jun Yang

**Affiliations:** 1 ShenSi Lab, Shenzhen Institute for Advanced Study, University of Electronic Science and Technology of China, Chengdu 518110, China; zhaoxiangyu1999@gmail.com (X.Z.); zhourong@nsccsz.cn (R.Z.); csningli@gmail.com (L.N.); guoqiuquan@uestc.edu.cn (Q.G.); 2 National Supercomputing Center in Shenzhen, Shenzhen 518005, China; 3 School of Resources and Environment, University of Electronic Science and Technology of China, Chengdu 611731, China

**Keywords:** atrial fibrillation, electrocardiogram, temporal convolutional network, residual network

## Abstract

Atrial fibrillation, one of the most common persistent cardiac arrhythmias globally, is known for its rapid and irregular atrial rhythms. This study integrates the temporal convolutional network (TCN) and residual network (ResNet) frameworks to effectively classify atrial fibrillation in single-lead ECGs, thereby enhancing the application of neural networks in this field. Our model demonstrated significant success in detecting atrial fibrillation, with experimental results showing an accuracy rate of 97% and an F1 score of 87%. These figures indicate the model’s exceptional performance in identifying both majority and minority classes, reflecting its balanced and accurate classification capability. This research offers new perspectives and tools for diagnosis and treatment in cardiology, grounded in advanced neural network technology.

## 1. Introduction

Atrial fibrillation, recognized as the most common persistent arrhythmia, is closely linked to increased risks of stroke, heart failure, and death [1]. Studies show a marked increase in the occurrence of atrial fibrillation with advancing age, highlighting its importance in cardiovascular health [2]. Beyond its prevalence, atrial fibrillation is a critical contributor to the morbidity and mortality rates in individuals with chronic arrhythmic conditions. Adding to the challenge is the often asymptomatic nature of this disorder, leading many to remain unaware of their condition until severe complications emerge. Therefore, the early identification and management of atrial fibrillation are of utmost importance.

**Figure 1:**
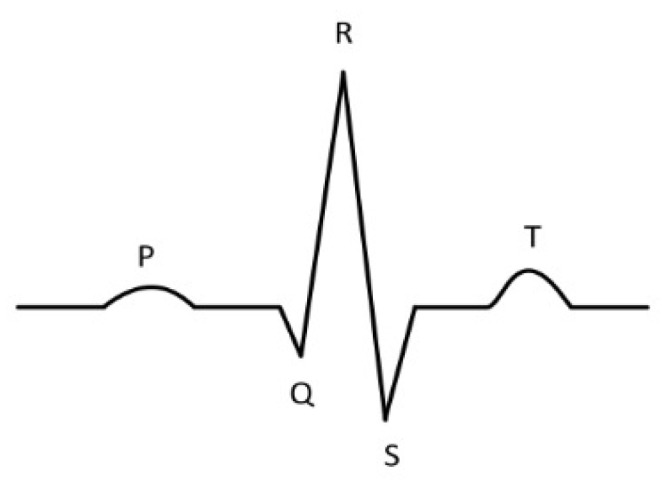
An ECG example.

In the realm of healthcare, electrocardiograms (ECG) are essential tools for medical professionals diagnosing atrial fibrillation. Under normal conditions, an ECG heartbeat clearly shows P waves, QRS complexes, and T waves, as depicted in Figure 1. However, in cases of atrial fibrillation, the characteristic P wave is absent, supplanted by erratic F waves that vary in direction and accompanied by irregular RR intervals [3]. Therefore, the development of effective algorithms for detecting atrial fibrillation is a critical endeavor, with significant implications for enhancing medical diagnostics and improving patient care outcomes.

The surge in electronic information technology has significantly expanded automated classification techniques for atrial fibrillation [4]. Generally, these methods follow a standard process that includes signal preprocessing, feature wave identification, heartbeat segmentation, feature extraction, and classification [5]. Feature extraction, particularly, is a critical step in traditional classification approaches, greatly affecting the performance of the algorithm and the final classification results. In traditional machine learning, manual extraction of features is essential to define ECG features in either temporal or spectral domains, requiring extensive medical knowledge and often lacking in generalization ability [6]. Conversely, deep learning approaches automatically derive features from the input data, leading to higher accuracy levels compared to manually extracted features [7]. Therefore, deep learning has increasingly become the preferred choice for ECG signal classification among researchers.

The contribution of this research lies in the significant enhancement of the ResNet model. We have achieved this by incorporating a pioneering network structure that merges the temporal convolutional network (TCN) with ResNet. This combination is particularly effective for extracting a wide range of features from various elements of the ECG signal. Our innovative approach leverages the TCN model to adeptly capture temporal characteristics, while the ResNet model is utilized for identifying features in the spectral domain. These diverse dimensions are then synergistically consolidated in the final fully connected layer of the network, which plays a crucial role in the classification of ECG signals.

The rest of the paper is organized as follows: Section 2 provides an overview of related works in the application of neural networks in atrial fibrillation classification. Section 3 describes the proposed method in detail. In Section 4, experiments are carried out and the results are analyzed. Finally, Section 5 concludes our research and offers insight into future research directions.

## 2. Materials and Methods

### 2.1. Related Works

In recent decades, the application of machine learning methods has seen widespread utilization in the domain of atrial fibrillation detection and classification. This research has led to the development of various algorithms, which have contributed significantly to enhancing the accuracy and efficiency of atrial fibrillation identification. This paper provides an overview of several noteworthy contributions in this field.

ChenWei Huang and JianJiun Ding [8] introduced a dual-layer strategy in their atrial fibrillation episode detection algorithm. This method encompasses the conversion of fundamental features into ratio-based representations, thereby emphasizing the intrinsic relationships between these features. Subsequently, they deployed a rule-based classifier that is specifically designed to focus on variations in these ratios. This classifier integrates multiple techniques, including the weighted coefficient function, product-form score function, Gini index, and Gini splitting function. After extensive training and testing on the MIT-BIH atrial fibrillation database, their algorithm achieved an impressive average sensitivity of 99.27% and an average specificity of 98.49%. Another significant contribution came from Rebeh Mabrouki and colleagues [9], who devised an innovative approach for atrial fibrillation detection. This approach incorporates two distinct nonlinear statistical methods—the Poincaré plot for evaluating RR interval series variability and sample entropy for analyzing the complexity of these series. Their algorithm was trained on the MIT-BIH atrial fibrillation database and tested on the MIT-BIH arrhythmia database, utilizing receiver operating characteristic (ROC)-curve-derived parameters. This effort yielded a sensitivity of 99.65% and a specificity of 99.33% when applied to the MIT-BIH atrial fibrillation database. Furthermore, it demonstrated a sensitivity of 97.91% and a specificity of 92.72% when tested on the MIT-BIH arrhythmia database. In a separate study, Resiandi and associates [10] employed the K-nearest neighbor algorithm to differentiate between atrial fibrillation and normal ECG readings, with a particular focus on the RR interval. Their research identified that the most effective configuration for atrial fibrillation detection in their scheme was when the parameter *k* equaled 1. This configuration resulted in an average accuracy of 91.75%, with a peak accuracy rate of 95.45% and a sensitivity of 91.67%.

In recent years, deep learning, represented by convolutional neural networks (CNNs), has achieved significant breakthroughs in various areas, including image classification, target detection, and semantic segmentation. CNNs possess the remarkable capability to automatically extract features from input data, thus alleviating the need for manual feature engineering [11]. They are also known for their robustness against noise and their ability to generalize well to diverse datasets. Therefore, CNNs have been effectively applied to feature extraction from ECG signals, emerging as one of the widely adopted methods in the field of ECG data analysis. Caiyun Ma [12] introduced an innovative method for atrial fibrillation detection that is suitable for non-hospital environments. This approach utilizes a support vector machine (SVM) enhanced by the predictive probabilities generated with a CNN. The model was trained using the MIT-BIH atrial fibrillation database, achieving impressive accuracies of 97.87% for 30 s ECG segments and 96.09% for 10 s segments through 5-fold cross-validation. When tested on the PhysioNet/Computing in Cardiology (CinC) Challenge 2017 database, the model demonstrated accuracies of 93.21% for 30 s episodes and 93.03% for 10 s episodes. Subsequent testing on the China Physiological Signal Challenge (CPSC) 2018 database yielded even higher accuracies of 98.48% and 98.61%,respectively. Additionally, the model’s generalizability was assessed by retraining it using the PhysioNet/CinC Challenge 2017 dataset and testing it on other databases, resulting in accuracies of 96.84% and 95.13% on the MIT-BIH atrial fibrillation database, 96.21% and 98.45% on the CPSC 2018 database, and 99.08% and 96.43% on wearable ECG datasets. In a distinct approach, Javid Farhadi and colleagues [13] applied a deep learning technique known as a stacked auto-encoder for differentiating atrial fibrillation ECG signals from typical ones. Their analysis involved ECG signals from the MIT-BIH database, and they focused on extracting spectral, temporal, and nonlinear characteristics. Initially, these extracted features were assessed through a statistical test, specifically the analysis of variance (ANOVA). Subsequently, the significant features identified through this process were applied to a stacked auto-encoder in a parallel arrangement to categorize atrial fibrillation and normal ECG samples. Brito et al. [14] studied the classification of electrocardiography (ECG) based on a ResNet architecture with convolutional 1D layers. The study includes experimental results using the MIT-BIH arrhythmia database. Ingolfsson and research team [15] present a novel, energy-efficient temporal convolutional network (TCN) for classifying ECG signals in wearable devices. Hu, Shaogang et al. [16] combined ResNet and TCN for ECG classification to extract both spatial and temporal features of ECG signals on the MIT-BIH dataset.

These contributions collectively underscore the remarkable progress made in the field of atrial fibrillation detection and classification through the integration of machine learning and deep learning techniques. However, most studies utilize multi-lead ECG recordings from a clinical setting, achieving high accuracy and efficiency. In contrast, single-lead ECG data collected from wearable devices exhibit unique challenges in terms of signal quality and data representation. The main difficulty lies in the limited information provided by single-lead ECG recordings, which can only capture a narrow snapshot of cardiac electrical activity. The restricted view makes it more difficult to detect a variety of cardiac abnormalities, which are more easily and accurately identified by multi-lead ECG recordings. Therefore, it it more challenging to develop reliable and accurate methods based on single-lead ECG recordings.

### 2.2. Background

#### 2.2.1. Temporal Convolutional Network

The temporal convolutional network (TCN), introduced by Bai et al. [17], represents a distinctive variant of convolutional networks tailored for time series data processing. It has shown superior performance over recurrent networks in various applications, including audio synthesis and machine translation. The TCN architecture comprises a 1D fully convolutional network (FCN) and dilated causal convolutions. The FCN ensures that the network’s output length matches the input length. Each hidden layer is designed to be of the same length as the input layer, with zero-padding of appropriate length added to maintain the consistent length across layers. Dilated causal convolutions are crucial in preventing the leakage of future information into the past. This design is achieved by altering the receptive field’s size through variable dilation coefficients, allowing the network to dynamically adjust the extent of historical data influencing the output. In the context of a one-dimensional input sequence *x* and a filter *f*, the receptive field’s scope can be expanded by adjusting both the filter’s coefficient *k* and the dilation coefficient *d*. The formula for dilated convolution is as follows:

(1) $$F\left(x\right)=\sum_{i=0}^{k-1}f\left(i\right)\cdot x_{s-d\cdot i}$$

**Figure 2:**
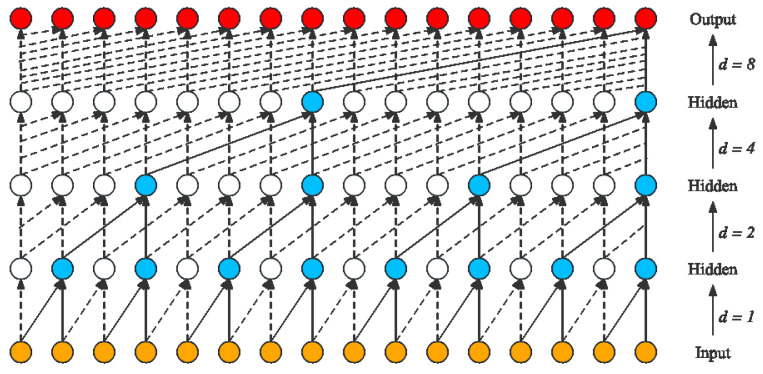
Dilated causal convolution structure.

In this equation, *d* represents the dilation coefficient, influencing the inclusion of historical data, denoted as s−d·i. The term *k* refers to the size of the filtering window. Figure 2 shows the a four-layer structure of dilated causal convolution.

#### 2.2.2. Residual Network

The residual network (ResNet), a groundbreaking model in deep learning, was proposed by Kaiming He [18] to address the challenges of vanishing and exploding gradients that are common in deep neural networks. Prior to ResNet’s introduction, a common approach to enhance model accuracy was to increase the number of layers in the network. However, this strategy often led to issues like network degradation and gradient vanishing, which paradoxically resulted in reduced network performance.

**Figure 3:**
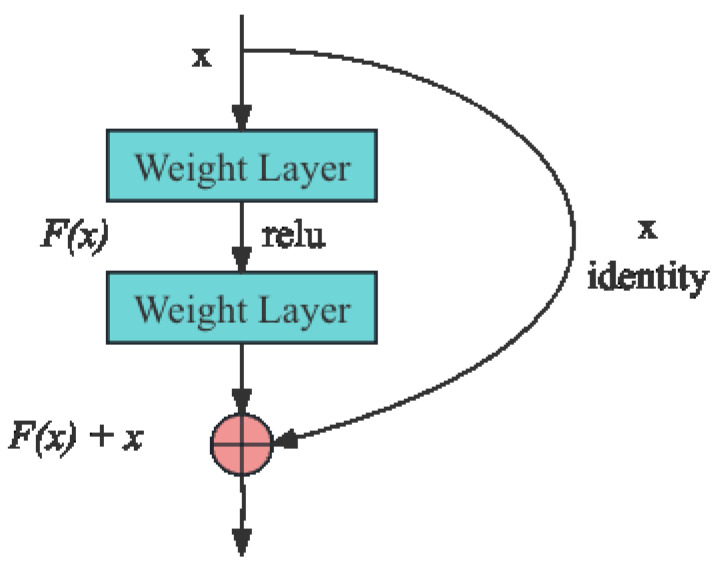
Residual network structure.

ResNet revolutionizes this approach by incorporating skip connections into the network architecture. As shown in Figure 3, these connections create shortcuts in the network, allowing for direct information transfer across layers. This design allows layers to learn residual functions with reference to the layer inputs, instead of learning unreferenced functions.

The shortcut connections in ResNet effectively mitigate the problem of vanishing gradients by facilitating the flow of gradients through the network. This allows for deeper networks without the associated degradation, as each layer can now pass its information directly to subsequent layers. This results in enhanced training efficiency and improved accuracy, even in networks with a large number of layers. The ResNet architecture has thus become a foundational model in deep learning, influencing the development of numerous subsequent neural network architectures.

### 2.3. Model Design

#### 2.3.1. TCN-ResNet

**Figure 4:**
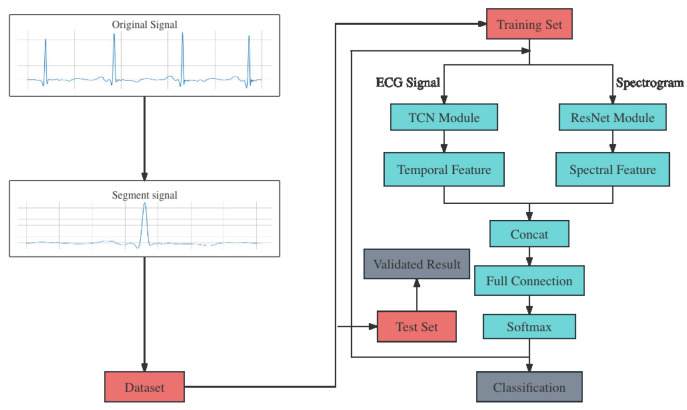
Algorithm framework.

Figure 4 shows an overview of the algorithm we proposed. It comprises four main steps: data preprocessing, dataset division, feature extraction and heartbeat classification. The progress begins with the segmentation of raw ECG signals from the original dataset. Then, the dataset is divided into training set and test set. In addition, spectrograms are calculated from the raw ECG signal, using the fast Fourier transform [19]. As a result, ECG singals and spectrograms are both input into the classification model, which belongs to the core part of the algorithm.

The model is composed of two distinct modules: TCN and ResNet. The TCN module receives ECG signals as its inputs and extracts the temporal features. Meanwhile, the spectrograms are input into the ResNet module and the spectral features are extracted. Then, the two different features are fused through concatenation. This step aims to combine different dimensions of information extracted from raw data, not only providing a more comprehensive representation of the data that enhances the model’s ability to capture its complexity but also improving the model’s generalization to new data through the complementarity of multiple features, thus reducing the risk of overfitting due to improper feature selection. Detailed information of these two modules are in the following section. The most common used PReLU activation function in this model are defined as follows:

(2) $$PReLU=\left\{\begin{array}{cc} x, & x>0\\ ax, & x\leq 0\;0<a<1 \end{array}\right.$$

PReLU is a variant of ReLU. It introduces a learnable parameter for negative input values, adaptively adjusting the shape of the activation function, thus enhancing the model’s flexibility and learning capacity in handling nonlinear issues. This characteristic allows PReLU to perform better than standard ReLU in complex and large scale deep learning tasks, especially where capturing finer data features is crucial.

#### 2.3.2. TCN Module

The core element of the TCN module, termed Temporal Block, is composed of the dilated causal conv layer, the weight normalization layer, the PReLU layer, and the dropout layer. Additionally, this block includes an optional convolutional residual connection, which enhances the network’s capacity for information transfer across layers and helps in reducing the issue of gradient vanishing. Within the same Temporal Block, the dilation factor for the convolutional layers is identical, and there exists a doubling relationship in this factor between successive Temporal Blocks.

**Figure 5:**
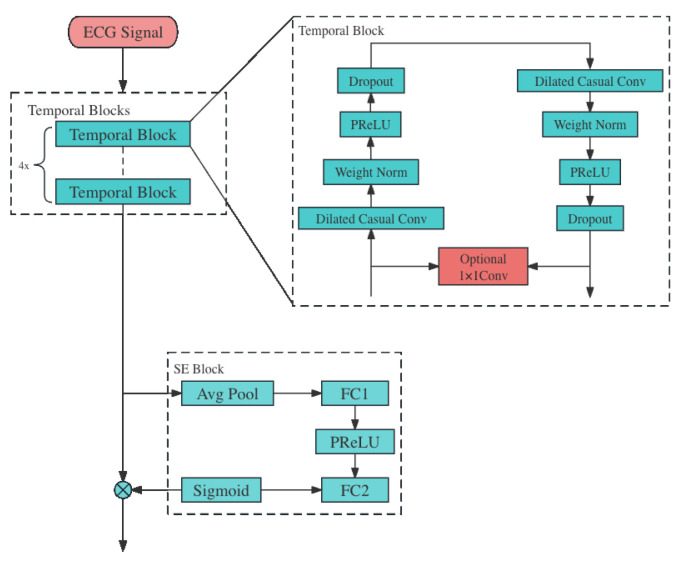
TCN Module.

The TCN module’s design, as illustrated in Figure 5, incorporates a structure of four Temporal Blocks. Each block is equipped with a convolution kernel size of 9, and the dilation factors for these kernels are sequentially set at 1, 2, 4, and 8 in the respective modules. This setup allows for an increasing field of view across the blocks. The quantity of convolutional kernels in each block escalates progressively, beginning with 128, then expanding to 256, 512, and ultimately reaching 1024. Throughout this progression, a consistent dropout rate of 0.2 is maintained to prevent overfitting. At the output of each block, the final node in every channel signifies the feature extracted by the network. These features represent the processed data at various scales, capturing both short-term and long-term dependencies in the input. Following these Temporal Blocks, the module integrates a channel attention mechanism, specifically employing the Squeeze-and-Excitation (SE) block. This component of the architecture plays a critical role in enhancing model performance. The SE block dynamically adjusts the weighting of different channel features according to the loss function. This adaptive focusing of the network’s attention allows it to emphasize more relevant features and suppress less important ones, leading to a more effective and nuanced understanding of the input data. The integration of the SE block is a strategic choice to bolster the network’s capacity to discern and prioritize pivotal information from the input sequence, thereby improving the overall efficacy of the TCN module. Table 1 shows the parameters of the major networks in this module.

#### 2.3.3. ResNet Module

**Figure 6:**
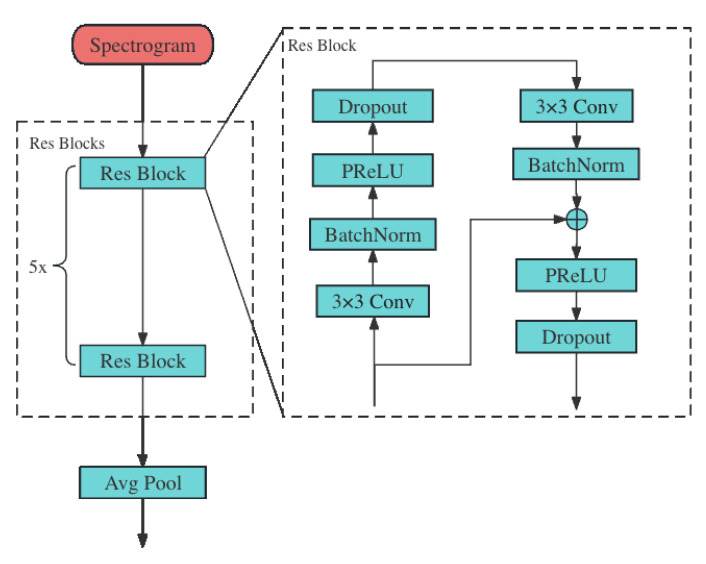
ResNet module.

Figure 6 delineates the structure of the ResNet module. It receives the spectrogram generated by the fast Fourier transform (FFT) of the raw ECG signal as input. Then, the spectrogram is passed to the Res Blocks to extract the spectral feature of the raw ECG signal.

Each Res Block comprises two residual layers, and each of these layers consists of four key components: the 2D convolution layer, the batch normalization layer, the PReLU layer, and the 2D dropout layer. The convolution layers within these blocks are characterized by a kernel size of 3×3 and a stride of 1×1, optimized for effective feature extraction. The spectral features extracted by these residual layers are subsequently downsampled using a 2D average pooling layer. This step is crucial for reducing the dimensionality of the data while retaining the essential information.

Moreover, the Res Block is notably enhanced by the implementation of a residual connection, which establishes a direct pathway between the input of the block and the batch normalization stage of the second residual layer. This design plays a crucial role in mitigating the vanishing gradient problem, a challenge that intensifies with increasing network depth. The residual connection not only ensures more efficient gradient flow during backpropagation, enabling deeper network architectures without compromising training effectiveness, but also facilitates the transfer of information from higher to lower levels within the network. This feature is especially beneficial in deep learning applications such as ECG signal processing, where it allows the network to preserve and leverage high-level feature information throughout the learning process, ultimately enhancing the model’s performance in detecting and analyzing cardiac signals. Detailed parameters of the major networks are shown in Table 2.

### 2.4. Dataset and Preprocessing

**Figure 7:**
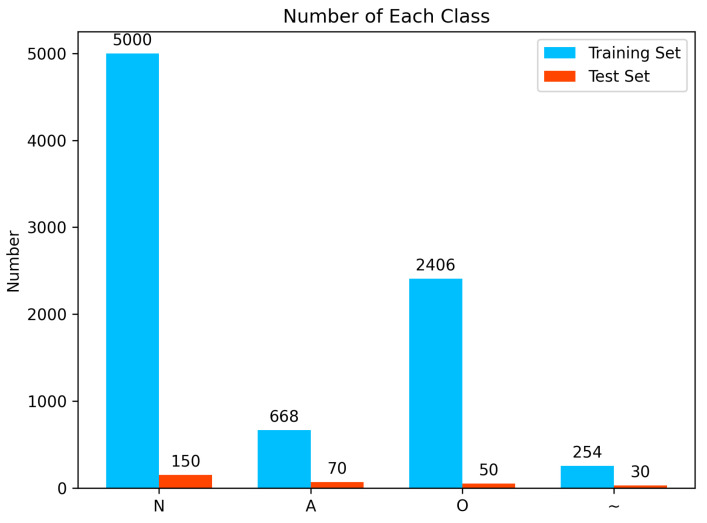
Number of each class.

In this work, we use the 2017 PhysioNet/CinC Challenge dataset [20], which is one of the most popular datasets for atrial fibrillation detection and ECG classification. This dataset contains over 8000 short single-lead ECG signals which are sampled with a frequency of 300 Hz and last from 30 s to just over 60 s. These ECG singals are classified into four classes: N for normal sinus rhythm, A for atrial fibrillation, O for other arrhythmias, and ~ for signals that are too noisy to classify. Then, the dataset is divided into a training set with 8228 records and a test set with 300 records. The number of each class in the dataset is shown in Figure 7.

The dataset studied exhibits notable class imbalance, with much less noisy data and atrial fibrillation compared to the other two categories. This may lead to model overfitting to majority classes during the learning process, making it difficult to effectively classify underrepresented classes. Also, in our study, we address the challenges posed by single-lead ECG data, which inherently have a lower accuracy and are more prone to noise compared to dual-lead data. This necessitates a robust model capable of handling noisy signals.

Inspired by the CutMix [21] technique in computer vision, we adapt this method to augment one-dimensional ECG signals. This technique involves selecting segments (time windows) from one ECG record and integrating them into another record. We choose variable-length time windows based on cardiac cycles rather than fixed lengths, ensuring that each segment represents a complete heartbeat or a meaningful part of the ECG cycle. In the insertion process, we meticulously align the segments to maintain the waveform’s continuity, ensuring no abrupt jumps or drops which could render the data clinically irrelevant or misleading. Each augmented signal is reviewed to confirm that the resulting ECG pattern remains plausible within a clinical context, preserving the integrity of potential diagnostic features. By augmenting underrepresented classes, such as noisy data, we increase their prevalence in the dataset. This enhancement not only balances the dataset but also introduces a wider variety of ECG patterns, critical for training a robust model. After data augmentation, we expand the number of the training dataset from 8228 to 9000.

The raw ECG signals in the dataset vary significantly in terms of voltage values, attributable to differences in equipment calibration, patient specific factors, and recording conditions. To standardize these signals and eliminate the impact of varying baselines, we employ Z-score normalization. This process transforms each ECG signal into a sequence with a mean of 0 and a variance of 1, thereby normalizing the data across all records. Following normalization, we convert the ECG signals into spectrograms using the fast Fourier transform (FFT). In our study, both time-domain and frequency-domain signals were input to different module of the proposed model, respectively. It is essential for identifying subtle yet clinically significant ECG features.

The final preprocessing step involves the segmentation of heartbeats from the padded signals. We adopt a fixed window size for this segmentation, typically encompassing a single cardiac cycle. This method allows the model to focus on individual heartbeats, thereby enhancing its ability to detect anomalies and irregularities within specific cardiac cycles. To address the issue of varying signal lengths, we implement zero-padding for signals shorter than the maximum length observed in the dataset. This ensures uniformity in input size for model training, without distorting the original information contained within the ECG signals.

Through the integration of these sophisticated data augmentation and normalization techniques, we address the critical issue of class imbalance in our dataset. This comprehensive preprocessing strategy not only ensures a balanced and representative training dataset but also enriches it with clinically relevant ECG patterns, significantly enhancing the model’s diagnostic accuracy and reliability.

### 2.5. Experiments

#### 2.5.1. Training and Validation

Our experiments were carried out on an Ubuntu 20.04.4 LTS system. We utilized Python 3.8.10 for programming, along with the numpy and Pytorch libraries for data handling and neural network operations. The hardware setup includes a 2.60 GHz Intel (R) Xeon (R) Platinum 8350C CPU, 56GB of RAM, and a GeForce RTX 3090 GPU with 24 GB of VRAM.

For model training, we set the epochs at 100 and initiated the learning rate at 0.0002. The network optimization was managed using RAdam, paired with a weighted cross-entropy loss function. Additionally, we implemented a learning rate scheduler to dynamically adjust the rate during training. To ensure robust training and test dataset selection and accurately reflect the algorithm’s performance, we incorporated a 10-fold cross-validation strategy.

#### 2.5.2. Evaluation Methods

For the evaluation of our model’s effectiveness in ECG signal classification, we utilize four standard performance indicators commonly employed in such studies. These are precision (P), recall (R), F1 score (F1), and accuracy (Acc). The computations for these metrics are based on the subsequent equations:

(3) $$P=\frac{TP}{TP+FP}$$

(4) $$R=\frac{TP}{TP+FN}$$

(5) $$Acc=\frac{TP+TN}{TP+TN+FP+FN}$$

(6) $$F1=\frac{2\times TP}{2\times TP+FP+FN}$$

In the formula, TP stands for the true positive, FP stands for the false positive, FN stands for the false negative, and TN stands for the true negative.

## 3. Results

### 3.1. Ablation Experiments

**Figure 8:**
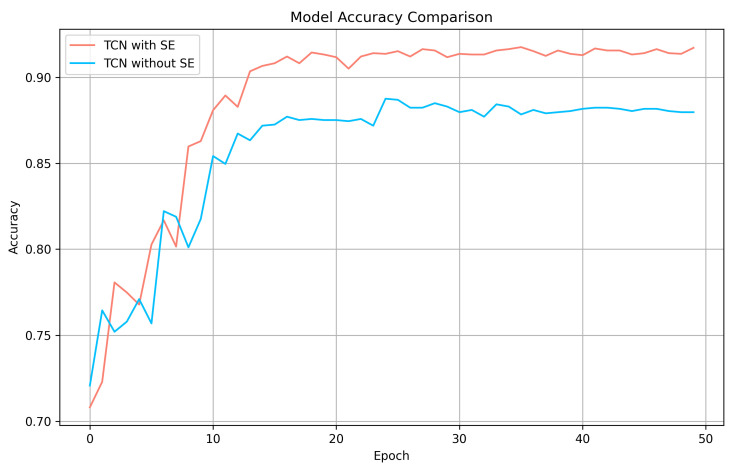
TCN model accuracy comparison.

At first, we verified the validity of the SE block in the TCN module. We conducted experiments on a TCN with SE blocks and another TCN with SE blocks replaced by Avg Pool. The accuracy comparison can be seen in Figure 8. As is shown in the figure, SE block effectively improves the accuracy of TCN module.

**Figure 9:**
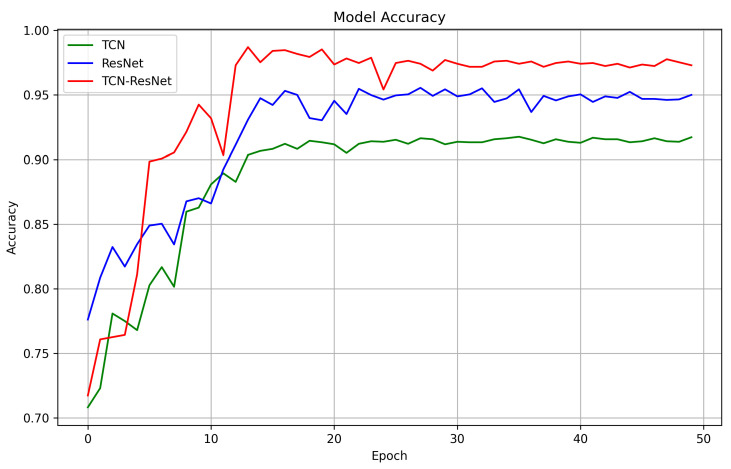
Comparison of accuracy on TCN, ResNet, and TCN-ResNet.

Next, we evaluated the respective contributions of TCN and ResNet in the combined model and the impact of their interaction on ECG classification. Specifically, a comparative analysis was conducted on the single TCN model, the single ResNet model, and the hybrid model combining TCN and ResNet. Table 3 and Figure 9 show the experiment results. We can see that as the model complexity increases, the classification performance becomes better. This is because TCN focuses on extracting 1D temporal features of ECG signals, while ResNet focuses on extracting 2D spectral features. Although the TCN model has effective performance in temporal feature extraction, it is inferior to the ResNet model in the overall classification task, and the spectral features extracted by the latter are more critical for distinguishing different classes of ECG signals. When the two are combined, the performance of the TCN-ResNet fusion model exceeds that of either individual model, indicating that the comprehensive extraction of temporal features and spectral features has significant advantages for the ECG four-classification task.

### 3.2. Comparison with Previous Work

In this section, we reproduce the work of multiple previous researchers and conduct experiments using the same dataset partition method as ours. The results are shown in Table 4. Our model demonstrates superior performance in identifying both the majority and minority classes, evidenced by its higher F1 scores for the minority class and overall. This indicates a balanced and accurate classification capability.

## 4. Discussion

The TCN-ResNet model represents a significant advancement in ECG signal analysis for atrial fibrillation detection. This study’s integration of TCN and ResNet architectures capitalizes on their respective strengths: TCN’s proficiency in capturing temporal dependencies and ResNet’s capability in feature extraction from complex signal patterns. The synergy between these two modules facilitates a more comprehensive analysis of ECG signals, enhancing the detection accuracy. Our model’s improved F1 score is particularly notable, as it reflects a balanced precision and recall ratio, crucial in medical diagnostics to minimize both false positives and false negatives. This balance ensures that the model is not only sensitive to the presence of atrial fibrillation but also specific in its detection, avoiding over-diagnosis and unnecessary medical interventions.

The implications of this study extend beyond the immediate results. The high F1 score achieved by the TCN-ResNet model opens possibilities for its integration into real-time monitoring systems, potentially operable in wearable devices. Such systems could provide continuous, non-invasive monitoring, offering significant benefits in patient care. Early detection of atrial fibrillation episodes allows for timely medical intervention, potentially reducing the risk of stroke and other heart-related complications. The use of a single-lead ECG in this model also points towards more accessible and less cumbersome monitoring methods, making it feasible for long-term patient monitoring in outpatient settings or even at home. This approach aligns with the current trend in healthcare towards more patient-centered and preventive care models.

While the current study demonstrates the TCN-ResNet model’s efficacy, future research should focus on further refining its performance. Enhancements could include improving the model’s sensitivity to variations in ECG signals caused by different physiological or pathological conditions. Additionally, expanding the model’s capabilities to detect other forms of cardiac arrhythmias could broaden its applicability, making it a more versatile tool in cardiac health monitoring. Another potential area of exploration is the model’s integration with other data types, such as patient history or biometric data, to provide a more holistic view of the patient’s health status. Finally, extensive clinical trials are essential to validate the model’s effectiveness in real-world settings, ensuring its reliability and accuracy in diverse patient populations. The ultimate goal is to integrate such advanced detection models into the healthcare system seamlessly, enhancing diagnostic procedures and patient care quality.

The results of this study have important implications for clinical practice and patient care. By combining a TCN and a ResNet, our method is able to detect atrial fibrillation more accurately and quickly, which is important for improving the patient outcomes are critical. Early and accurate diagnosis of atrial fibrillation can significantly reduce the risk of stroke and other complications, as this allows doctors to take timely preventive measures. The development of this technology may also provide opportunities for remote monitoring and long-term heart health management, opening up new possibilities, especially for patients who live in remote areas or do not have immediate access to specialized medical facilities. In conclusion, our study highlights the potential applications of using advanced neural network techniques in the field of cardiology, providing insights into clinical practice and a new, effective tool for detecting atrial fibrillation.

## 5. Conclusions

In our research, we innovatively combined TCN and ResNet into a cohesive ECG classification model, uniquely exploiting ResNet’s ability to extract frequency characteristics and TCN’s proficiency in time domain analysis. This synergy significantly elevates classification efficiency, especially compared to the approaches in “Electrocardiogram Beat-Classification Based on a ResNet Network” and previously discussed articles, which focus either on ResNet or TCN independently. Validating our model against the challenging 2017 PhysioNet/CinC dataset, we successfully demonstrated its capability in addressing the notable class imbalance through a bespoke data augmentation strategy, inspired by CutMix yet carefully adapted for ECG time series data. This strategy, alongside our innovative TCN-ResNet model, led to superior F1 scores, underscoring our method’s advanced proficiency in detecting atrial fibrillation from single-lead ECG signals. Future research will concentrate on further refining the model’s metrics and broadening its scope to encompass a wider range of cardiac arrhythmias, emphasizing its potential in clinical applications.

## Figures and Tables

**Table 1:** Parameters of Major networks in TCN module.

Block	Network	In Channels	Out Channels	Dilation Factor
Temporal Block 1	1 × 9 Conv	1	128	1
	1 × 9 Conv	128	128	1
	1 × 1 Conv	1	128	—
Temporal Block 2	1 × 9 Conv	128	256	2
	1 × 9 Conv	256	256	2
	1 × 1 Conv	128	256	—
Temporal Block 3	1 × 9 Conv	256	512	4
	1 × 9 Conv	512	512	4
	1 × 1 Conv	256	512	—
Temporal Block 4	1 × 9 Conv	512	1024	8
	1 × 9 Conv	1024	1024	8
	1 × 1 Conv	512	1024	—
SE Block	Full Connection	1024	512	—
	Full Connection	512	1024	—

**Table 2:** Parameters of major networks in ResNet module.

Block	Network	In Channels	Out Channels
Res Block 1	3 × 3 Conv	1	128
	3 × 3 Conv	128	128
Res Block 2	3 × 3 Conv	128	256
	3 × 3 Conv	256	256
Res Block 3	3 × 3 Conv	256	512
	3 × 3 Conv	512	512
Res Block 4	3 × 3 Conv	512	512
	3 × 3 Conv	512	512
Res Block 5	3 × 3 Conv	512	256
	3 × 3 Conv	256	256

**Table 3:** Ablation experiment results.

	Accuracy (%)	Recall (%)	Precision (%)	F1 Score (%)
TCN	91	80	85	82
ResNet	95	82	89	84
TCN + ResNet	97	92	92	87

**Table 4:** Performace comparison with related works.

Model	Accuracy	Precision	Recall	F1 Score				
				**N**	**A**	**O**	**~**	**Overall**
Andreotti [22]	89	75	74	88	67	66	65	72
Hong [23]	92	84	86	92	86	80	81	85
Christoph [24]	96	88	88	88	92	76	81	84
**TCN-ResNet (Our model)**	97	92	92	92	93	81	82	**87**

## Data Availability

The dataset used in this paper is a public dataset, and it can be downloaded from https://www.physionet.org/content/challenge-2017/1.0.0/ (accessed on 14 November 2023).
